# Establishing the foundation for technology adoption: profiles of military students in the digital age

**DOI:** 10.3389/fpsyg.2025.1593326

**Published:** 2025-05-23

**Authors:** Andrea Luna, Mauro Ocaña, Javier Rodríguez-Moreno, Ana María Ortíz-Colón

**Affiliations:** ^1^Universidad de las Fuerzas Armadas ESPE, Sangolquí, Ecuador; ^2^University of Jaén, Jaén, Spain

**Keywords:** latent profile analysis, MSLQ, learning profiles, educational technology, military students, self-regulated learning

## Abstract

Military students are often believed to possess different traits compared to civilian students, e.g., discipline and motivation. Yet, to implement a technology-enhanced learning environment, stakeholders must first address the complexity of student learning in military higher education institutions. Through a mixed-methods approach, we used latent profile analysis of MSLQ scores, complemented by a comprehensive study of academic programs and faculty interviews, to reveal that heterogeneity among military students is not significantly different from that of civilian students. We identified four distinct groups: (1) highly motivated students with strong self-efficacy and learning strategies, (2) students with low self-efficacy and high anxiety, (3) moderately motivated students, and (4) students with inconsistent learning profiles. Our analysis revealed that while the current pedagogical structure aims to balance theoretical, practical, and autonomous learning, it may not effectively meet the needs of all student profiles. However, LMS platforms, military simulators, and other digital tools can serve not only as instructional resources but also as potential catalysts for truly personalized and adaptive military education. Effective military pedagogy in the 21st century must recognize and address the diversity of learning profiles. This includes strengthening self-regulation and cognitive strategies through tailored technological interventions, optimizing digitally enriched hands-on activities, and increasing teacher awareness of motivational and emotional factors. This research offers a concrete roadmap for optimizing military training, positioning technology as a key driver of pedagogical transformation to better prepare future military leaders for the challenges of an ever-evolving world.

## 1 Introduction

In an era where Extended Reality (XR) and Human-Computer Interaction (HCI) are ubiquitous in educational environments, several student traits play a key role in successful learning. These traits are particularly relevant in Immersive Virtual Reality (IVR) environments which require students to exercise autonomous learning to either complete technical tasks or make additional efforts to adapt to novel interfaces. These traits are frequently observed in (1) self-efficacy, which influences how students approach challenges and their persistence in learning; (2) intrinsic value, which reflects the importance students assign to a task, their personal interest and motivation; and (3) self-regulation, understood as the ability to manage one’s learning processes, including goal-setting, the application of learning strategies, as well as the regulation of behavior, encompassing both emotional and cognitive dimensions (Cheng and Tsai, 2020; Romero-Ayuso et al., 2020).

To better understand students’ behavior toward the use of technology, it is essential to analyze their self-motivation, self-efficacy and self-regulation in the cognitive learning process. Equally important is the evaluation of virtual laboratories as comprehensive learning systems (Estoque Loñez and Errabo, 2022). The same applies to other technologies such as AI-based applications, chatbots, virtual coaches, and other immersive technologies (Bhange et al., 2024; Ocaña et al., 2023). These technologies demand a broad range of cognitive self-regulation skills (self-efficacy), emotional intrinsic value, and behavioral adaptability (responses to various contexts), which are key indicators of well-being (Selaskowski et al., 2023).

Since students are not all the same, it is important to identify their strengths to take advantage of technologies. For instance, in Northern European countries, students are generally expected to have a strong sense of self-efficacy, in line with the principles of the Scandinavian and *German Didaktik* concept, which emphasizes the relationship between content, student and teacher (Vallance, 2021). The influence of technology on students depends on several factors, e.g., location (Jusoh et al., 2021; Ocaña et al., 2021). Research suggests that integrating self-regulated learning strategies in immersive virtual reality environments positively impacts self-efficacy and perceived cognitive load by improving academic performance, metacognitive awareness, self-assessment efficacy, and time management. This enables students to self-evaluate by adjusting their expectations and efforts accordingly (Wu et al., 2021).

The growing interest in effectively incorporating immersive technology into educational context at all levels (Quinga et al., 2022; Vega et al., 2022) is evident in research on Virtual Reality (VR) and Augmented Reality (AR) in self-regulated learning. Countries such as Canada, the United States, Denmark, Indonesia and Spain have made significant progress in integrating these technologies into the pedagogical environment (Luna-Guillén et al., 2023). But there are still some challenges, one of which is test anxiety, the degree of nervousness students experience during exams. While certain video games, e.g., *Habitica*, can foster a sense of agency and promote learning, their effectiveness may be limited in high-pressure situations (Madera and Figueroa, 2019). Some research-based interventions have aimed to alleviate this pressure. For instance, *Anxiety Avatars* function as extensions of a user’s identity, enabling the integration of teaching strategies that promote mental well-being and significantly reduce anxiety levels (Pimentel, 2019).

The connection between self-regulated learning and technology has become increasingly evident in recent years. During the COVID-19 pandemic, the need to optimize self-regulated practices became more urgent, leading to proposals that integrate internal factors— such as cognitive skills, learning strategies, and motivation – with external contextual factors, including social, cultural, and technological environments (Dumulescu et al., 2021). Since then, multimodal data – such as verbalized thought protocols, physiological responses, and motion analysis – have proven valuable in understanding metacognitive monitoring and cognitive load dynamics in learners which has provided insights into self-regulated learning processes in immersive virtual reality environments (Sobocinski et al., 2024).

Students who purposefully engage with technology for study-related activities achieve higher academic success (Juuti et al., 2022). So, given the strong link between self-regulated learning and technology, its optimization depends on instructional supports such as structured practice schedules, normative comparisons, and clearly defined learning. These should be implemented without neglecting motivation and time management, both of which maximize skill retention and transfer in real-world contexts (Cook et al., 2019). In this regard, distance learning has demonstrated significant academic advances through the use of educational software, technology platforms, radio and the Internet (Rosyadi et al., 2021; Nee et al., 2022).

Based on this context, the objective of this study is to evaluate the level of self-regulated learning and the use of immersive technologies in the context of military education, identifying the key components necessary for the optimal use of technology.

## 2 Materials and methods

This study, which is part of a project approved by the Chief of the Joint Command of the Armed Forces, achieved a response rate of 45.85% and employed a mixed-methods approach, following a sequential explanatory design. In a first phase, the MSLQ questionnaire (Pintrich and Schrauben, 1992) was administered to 619 students from military institutions to collect quantitative data on self-regulated learning. The MSLQ assesses student motivation and learning strategies, focusing on two key components: motivational beliefs (self-efficacy, intrinsic value and test anxiety) and self-regulated learning (cognitive strategies and self-regulation). It uses a 7-point Likert scale to measure 44 items (Panadero, 2017; Pintrich and Schrauben, 1992).

These data were subjected to Latent Profile Analysis (LPA), which involves identifying hidden subgroups of students with similar learning features. While previous studies have demonstrated the usefulness of clustering subgroups of individuals with similar characteristics in learning environments (see, e.g., Ocana et al., 2019; Guallichico et al., 2023), LPA is a person-centered approach that probabilistically models each individual’s likelihood of belonging to a profile. This means that LPA preserves individual differences and can suggest actionable insights for academic interventions (Ferguson et al., 2020).

Subsequently, a documentary analysis of the institutions’ curricula was conducted to compare the data obtained from the questionnaire. This analysis focused on identifying key components of self-regulation, including self-efficacy, intrinsic task value, motivation, and cognitive strategies. Additionally, the curricular structure was examined to identify three fundamental types of learning: teacher-led learning, autonomous learning and experiential learning, as well as their relationship with the self-regulation strategies reported by students and lecturers.

In addition, an exhaustive review of the specialized literature, along with the operationalization of the variables, Self-Regulation of Learning and Immersive Technology. This process enabled the conceptual definition of the variables, and the development of an interview guide for 12 lecturers from the participating institutions.

Triangulation of the test results, interviews and curricular analysis helped to identify gaps between pedagogical practices and institutional guidelines, as well as strategies to enhance self-regulated learning through immersive technologies. For the analysis of quantitative data, Python was used to apply statistical tests aimed at identifying profiles of students with similar characteristics. The optimal number of profiles was determined by selecting the solution with the lowest AIC, BIC values along with the highest entropy. Initially, profile solutions ranging from 2 to 6 were assessed and the 4-profile solution was found to yield the lowest AIC (2450.3) and BIC (2520.8) values, as well as the highest entropy (0.91). To ensure stability and statistical rigor, a parametric bootstrap analysis was conducted (see Efron and Tibshirani, 1994). Using the same sample size (*n* = 619), 1,000 iterations were run, confirming the 4-profile solution in 93% of the cases which indicates a high stability.

In line with our mixed-methods research design, both quantitative and qualitative data were gathered. For the quantitative component, the MSLQ was administered in six military schools of the Armed Forces. The data were then cleaned and checked for noise and null values. In addition to the MSLQ, study programs and semi-structured interviews were also included. Following the Explanatory Sequential Design — which uses qualitative data to explain quantitative findings (Creswell and Creswell, 2018, pp. 213–246), — we: (1) aligned quantitative subscales from the MSLQ with (2) qualitative codes during CAQDAS analysis, and (3) mapped curricular learning components (autonomous learning, experiential learning, teacher-led learning) to the identified profiles. This procedure enabled clearer cross-validation across the three data sources, thereby strengthening integration.

For qualitative data, we used criterion sampling to gain deeper insights (Patton, 2014). As profiles were distributed on the six military schools, and to meet the objectives of our research, study programs from the six schools were analyzed to establish connections between syllabus content and MSLQ subscales. To differentiate these study programs, unique identifiers were assigned to each according to the three military branches (represented by their Spanish initials: E, A, M) and the level of seniority, represented by O and V.

To triangulate information analyzed hitherto, 12 lecturers were selected for semi-structured interviews, following the principle of data saturation (Saunders et al., 2018). Thematic analysis was applied to the qualitative data, using MSLQ components—self-efficacy, intrinsic value, test anxiety, cognitive strategies, and self-regulation—as the primary analytical framework. The analysis of interviews and curricular content was conducted using a CAQDAS (Computer-Assisted Qualitative Data Analysis Software), which facilitated the coding, categorization and subsequent understanding of how the qualitative information related to the previously identified profiles.

Next, results are presented in an integrated order: first, the MSLQ profiles; then, the analysis of study programs (highlighting aspects not captured by the MSLQ); and finally, findings from the semi-structured interviews, used to triangulate insights and provide a more holistic picture (Carter et al., 2014).

## 3 Results

**Figure 1:**
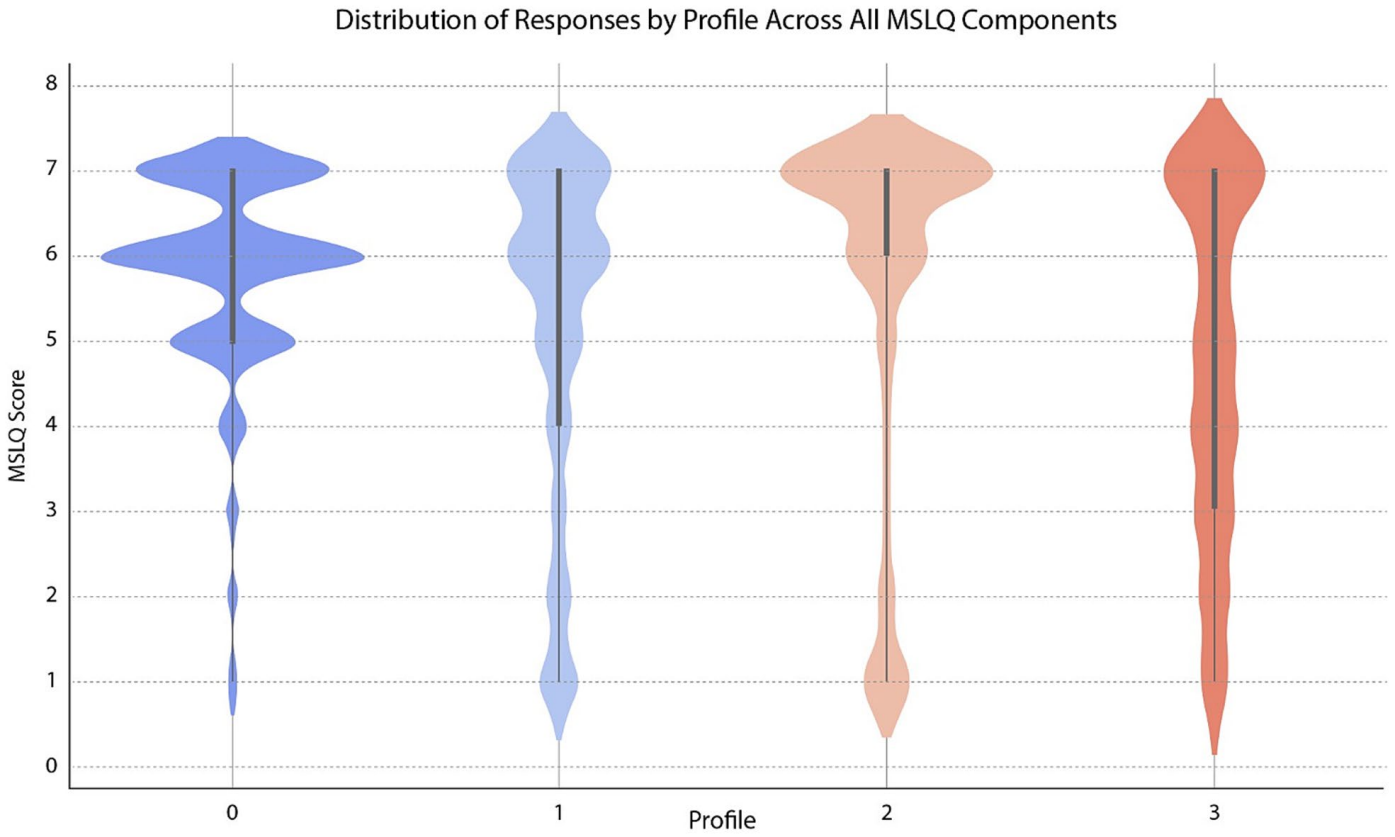
Distribution of responses by profile across all MSLQ components.

The distribution of responses by profiles across all MSLQ components is shown in Figure 1.

### 3.1 Profile 0 (blue): highly motivated

This group exhibits a high level of self-efficacy, particularly in items measuring self-efficacy through peer comparisons, which means that this group believes in their ability to perform well. They perceive tasks as relevant which is reflected in their high intrinsic value scores, demonstrating a strong connection between academic content and personal interests. Their low anxiety levels suggest they can effectively regulate their emotions during tests. Moreover, they excel in self-regulation by employing effective organizational strategies and information management techniques. They demonstrate persistence even when learning resources are difficult to navigate.

This aligns with their high self-regulation levels, as they remain committed to learning even in challenging situations. Additionally, this group of students use strong cognitive strategies which indicates effective study habits. In general, this group of students is highly motivated, exhibit strong learning strategies and do not display any weaknesses in their scores. For example, one of their highest self-efficacy scores (confidence in learning, see section 1) was the item “I expect to do well compared to others” where they achieved an average of 6.40.

### 3.2 Profile 1 (light blue): moderately motivated

This group shows characteristics similar to those of Profile 0 but exhibits slightly lower self-efficacy. Their interest in the task is moderate which suggests a weaker intrinsic connection than Profile 0. They experience moderate anxiety, with spikes among students who feel less confident in their preparation. They use cognitive strategies less frequently than Profile 0. While they perform well in self-regulation, some inconsistencies appear in their long-term planning.

### 3.3 Profile 2 (light coral): low self-efficacy-high anxiety

This group typically has lower scores, which reflects a general lack of self-confidence. Their interest in tasks is low, indicating a lack of intrinsic motivation for learning. Their high test anxiety scores indicate strong concerns during exams. Similarly, their cognitive strategies scores are low, which reflects difficulties in applying these strategies to learning. The same pattern is observed in their self-regulation scores. This suggests ineffective self-regulation and poor cognitive strategies, as they struggle to apply effective learning methods. This group also demonstrates low self-regulation, struggling to stay focused and motivated when faced with challenging learning materials. Their high test anxiety during exams may negatively impact their performance.

### 3.4 Profile 3 (salmon): inconsistent

This group of students exhibits high variability, with some students displaying confidence while others appear hesitant and less confident. Similarly, students’ perceptions of task value vary widely, with some finding tasks meaningful while others do not. Their anxiety levels fluctuate which suggests individual differences in emotional regulation. Cognitive strategy use is inconsistent, with students applying these strategies irregularly. Similarly, they exhibit significant variability, alternating between self-regulated behaviors and periods of academic disengagement (Table 1).

**Table 1:** Profile classification based on MSLQ results.

MSLQ component	Profile 0 (blue)	Profile 1 (light blue)	Profile 2 (light coral)	Profile 3 (salmon)
Self-efficacy	High	Moderately-high	Low	Inconsistent
Intrinsic value	High	Moderate	Low	Variable
Test anxiety	Low	Moderate	High	Inconsistent
Cognitive strategies	High	Moderate	Very low	Irregular
Self-regulation	High	Moderate	Very low	Irregular

### 3.5 Comparison of components across profiles

**Figure 2:**
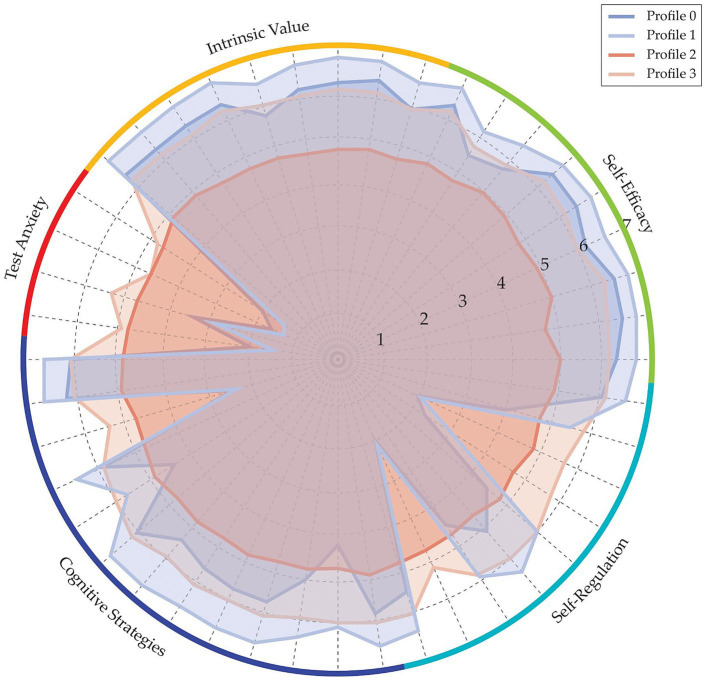
Distribution of MSLQ component scores across profiles.

The MSLQ components across profiles are shown in Figure 2.

## 4 Study programs

**Figure 3:**
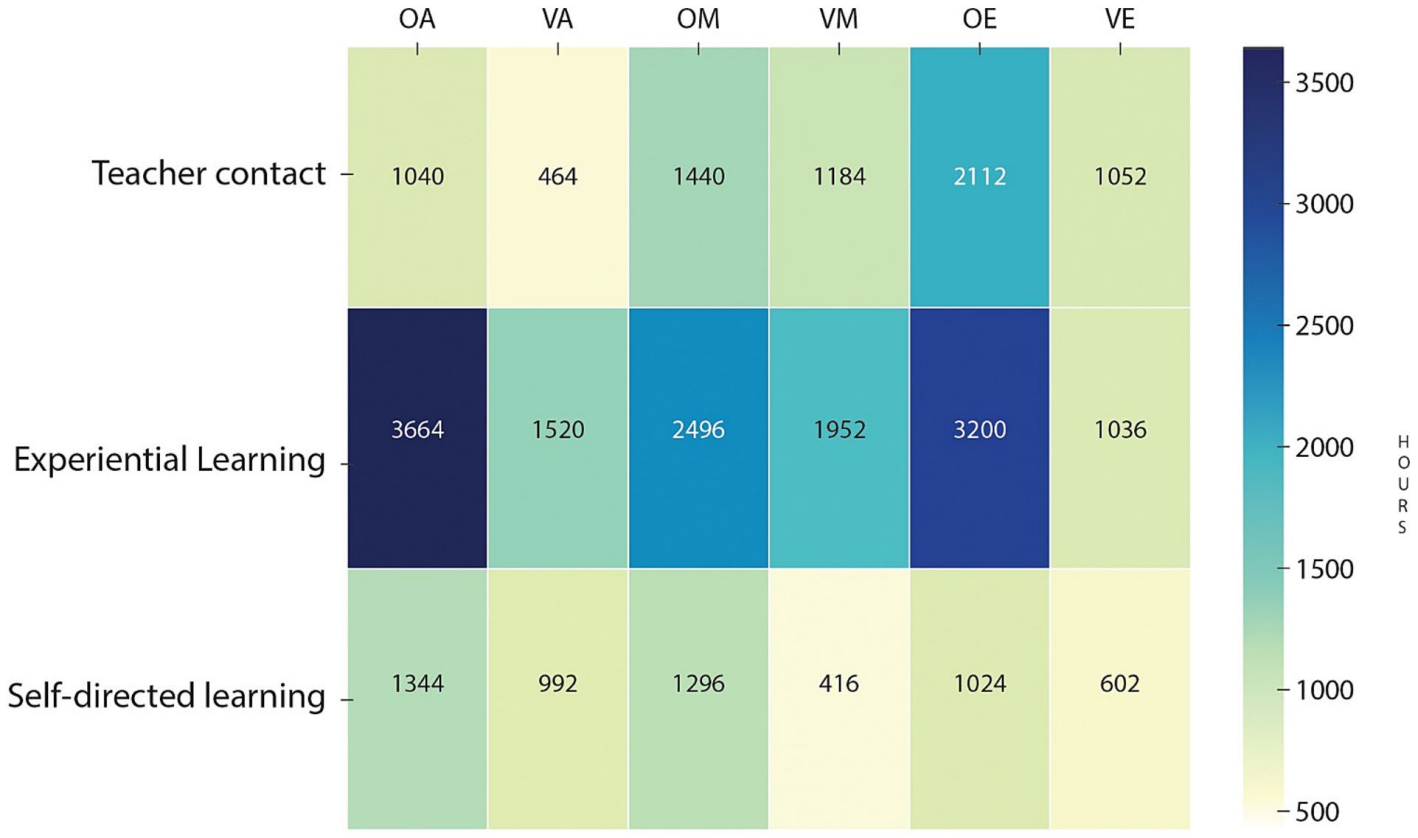
Heatmap of learning components in study programs.

Study programs in military training institutions incorporate key learning components designed to promote a holistic learning experience. These components include Teacher Contact (TC), which focuses on direct interaction between teachers and students; Experiential Learning (EL), which uses activities based on simulations, projects, and real-world fieldwork; and Autonomous Learning (AL), which promotes independent study. Together, they integrate theory, practice and self-directed learning. This framework aligns with the specific characteristics and objectives of each academic program, fostering the development of self-regulated learning (Figure 3).

The allocation of learning hours differs significantly across student groups. Most groups dedicate the largest portion of their hours to Experiential learning reflecting a preference for hands-on activities and real-world scenarios. For example, the OA group spends a substantial amount of time on experiential learning, closely aligning with their specialized training needs. Similarly, the OM group also has a heavy workload in this component, emphasizing the significance of practical experience in their field. Autonomous learning remains at a moderate level across all groups. However, it is more prominent in the OA group, indicating a greater emphasis on independent study. Finally, teacher contact varies among groups, with the highest levels observed in the OE group, possibly due to a greater need for direct guidance in their learning process.

This distribution reflects how each group structures its learning process based on specific needs and goals, fostering both independence and deeper engagement.

## 5 Interviews

**Figure 4:**
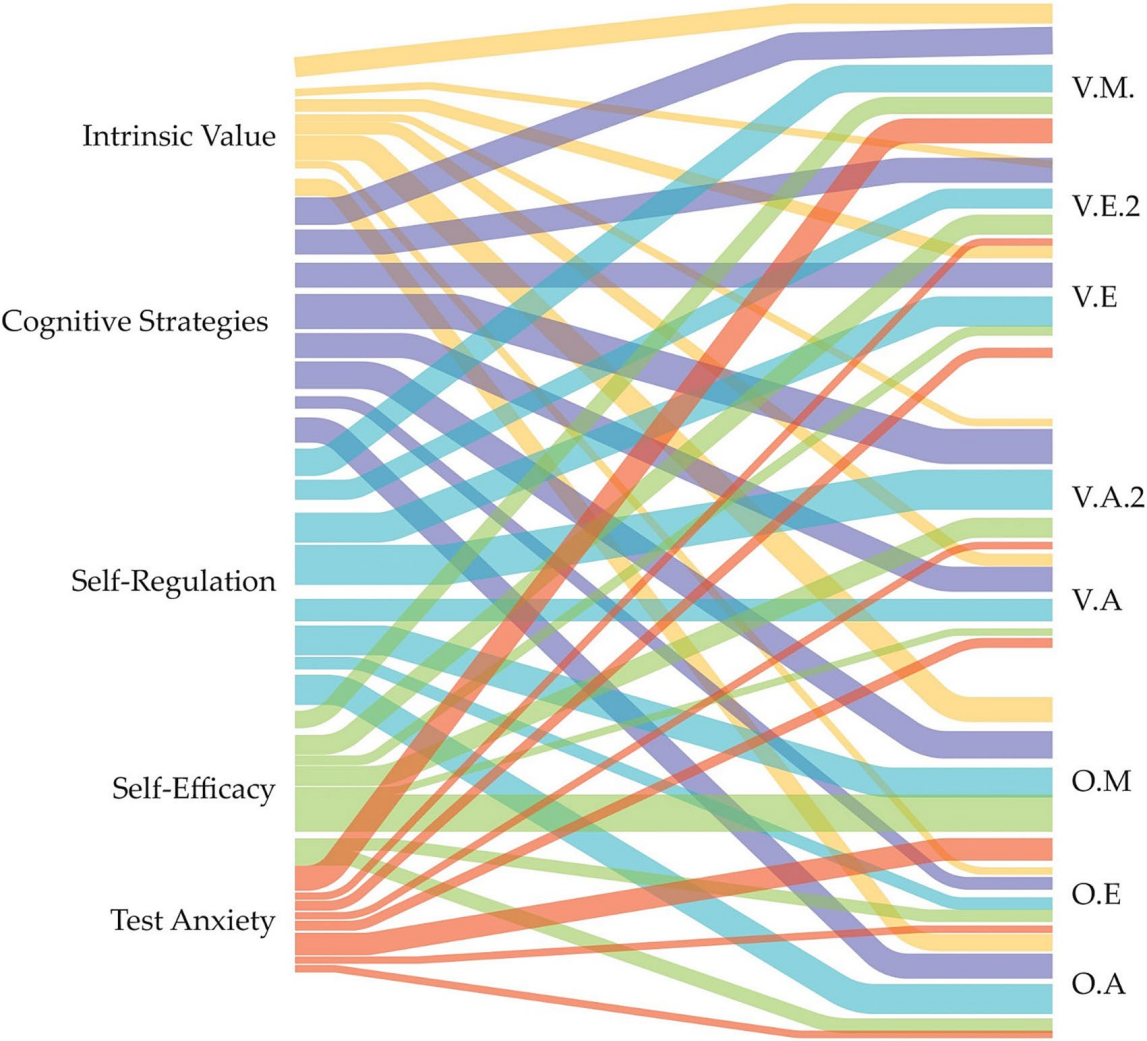
Sankey diagram, relationship and frequency of lecturers’ perceptions from military institutions.

The interviews, conducted with civilian and military lecturers from the participating military institutions, gathered perceptions on key factors such as test anxiety, self-efficacy, self-regulation, cognitive strategies, intrinsic motivation, and the intrinsic value of tasks. These insights provide a qualitative representation of lecturers’ assessments (Figure 4).

Interview responses were categorized based on key dimensions, with self-regulation (96 mentions) and cognitive strategies (88 mentions) emerging as the most frequently referenced themes. Conversely, test anxiety and intrinsic motivation had the fewest mentions. Notable consistencies were found across the analyzed dimensions, particularly in the relationship between self-regulation and cognitive strategies. Moreover, planning and technology integration play a crucial role in the teaching process, supporting strategies such as summarization, concept mapping, and structured exercises, which help manage large amounts of information and reinforce autonomous learning.

The intrinsic value of homework significantly contributes to the formative process in schools (VA, OA, VT), as it strengthens the connection between academic content and real-world experiences through practical exercises and real-case analysis, reinforcing the practical applicability of knowledge. Military lecturers emphasize the importance of instilling in cadets a mindset of ‘mental toughness’ and self-leadership. According to them, students’ ability to self-regulate through personalized feedback (OM), manage their emotions, and balance academic responsibilities within a highly disciplined environment is crucial to their success.

Although intrinsic motivation appears at a low percentage in the analyses, interviews highlight that teaching breathing techniques, self-control, and resilience in high-pressure situations significantly enhances emotional self-regulation (EO). Digital learning platforms such as Moodle, Google Classroom (OE), and other LMS are widely used for instruction and academic data management. Additionally, artificial intelligence and immersive technologies, including flight simulators and mechanical training, are widely implemented in VA and VE schools. However, in VE, limitations exist in the use of basic laboratories focused on physical simulations. Slight differences were observed in test anxiety levels; however, in OM and VM, academic pressure was identified as a key factor in developing mental resilience. Although social interactions were not a major focus, interviews in OA emphasized the importance of self-leadership and teamwork in managing academic and military responsibilities effectively.

Self-efficacy plays a crucial role in academic performance and students’ confidence in their abilities (OM, VM, VE). Lecturers highlight that developing skills such as planning, decision-making, and responsibility management strengthens cadets’ sense of competence, enabling them to tackle academic and military challenges more confidently.

## 6 Triangulation

### 6.1 Key similarities

Common findings across the MSLQ results, curricula, and interviews include high self-efficacy, averaging 6.2 out of 7, and moderate self-regulation, averaging 4.9 out of 7. To address these findings, curricula incorporate training in leadership, decision-making, and self-learning—key elements in strengthening self-regulation.

Regarding cognitive strategies, some students use tools such as concept maps and summaries; however, their use is not widespread. Teachers report implementing synthesis strategies and autonomous learning methods supported by technology, which has encouraged the adoption of active methodologies in curricula, such as Problem-Based Learning (PBL) and simulations.

Students who use digital tools tend to perform better in self-regulation. Lecturers highlight the significance of platforms such as Moodle, Google Classroom, and Virtual Reality simulation tools, though they acknowledge technical limitations that affect their effectiveness.

### 6.2 Differences and contrasts

Intrinsic motivation in task performance is not a strength among the evaluated students. Teachers note that military pressure impacts students’ motivation. Some use resilience-building and positive reinforcement techniques, while others prioritize strict discipline. Motivation is not explicitly addressed in the curricula, as they primarily emphasize compliance and discipline.

The MSLQ does not directly measure the impact of immersive technologies; however, it does indicate the level of self-regulation required for their adoption. Interviewed lecturers noted that not all institutions have incorporated tools such as Virtual Reality (VR), combat simulators, or AI-assisted learning due to limitations in infrastructure and Internet access. As a result, some schools continue to rely on traditional methods.

The academic load, combined with physical and cognitive training demands, poses a challenge for students, requiring them to manage both study and military training. Lecturers report that high physical demands occasionally interfere with academic performance. In some military institutions, military instruction occupies more time, reducing opportunities for autonomous learning. The significance of self-regulation is evident, with technological and cognitive strategies employed to enhance learning. Teachers promote self-regulation through innovative methods such as real-case analysis, problem-based learning, and digital tools. These practices strengthen curricula by enabling the monitoring and tracking of academic progress.

However, despite the implementation of these strategies, challenges related to student motivation persist. While immersive technologies and digital tools provide numerous advantages, disparities in access continue to hinder their equitable application in the learning process.

## 7 Discussion

This research employed a mixed-methods approach to analyze the student traits essential for effectively adopting technologies. Through Latent Profile Analysis (3.1), study program analysis (3.2), and lecturers’ perspectives (3.3), we gained a comprehensive understanding of these traits. This section integrates and discusses key findings from each phase, revealing significant patterns and highlighting implications for military educational practice.

Latent Profile Analysis (3.1) identified four distinct profiles of military students, differentiated primarily by motivation, self-efficacy, test anxiety, cognitive strategies, and self-regulation. Profile 0 (Highly Motivated) is characterized by high self-efficacy, intrinsic value, low anxiety, and effective use of cognitive strategies and self-regulation. Profile 1 (Moderately Motivated) exhibits similar traits but at slightly lower levels. In contrast, Profile 2 (Low Self-Efficacy - High Anxiety) lacks confidence, exhibits low intrinsic motivation, experiences high anxiety, and struggles with cognitive strategies and self-regulation. Finally, Profile 3 (Inconsistent) demonstrates significant variability across all dimensions, indicating a heterogeneous group with irregular learning patterns.

The analysis of their curricula (3.2) contextualizes these profiles by outlining a pedagogical framework composed of the Teaching (CD), Experimental Practical (CPE), and Autonomous (CA) components. These components are adapted to different military branches (VE, OA, OM, OE, VA, VM) to balance theory, practice, and autonomy within each specialization. However, the effectiveness of this structure may vary depending on the student’s profile.

For example, students in Profile 2, who exhibit low self-regulation, may struggle with the Autonomous Component (AC), which is designed to promote independent learning. It is important to recognize that student performance in digital environments depends on the interaction between Socially Shared Regulation of Learning (SSRL) and Self-Regulation of Learning (SRL). SSRL involves joint goal management, planning, and monitoring, which promotes collaboration and problem-solving. SRL, on the other hand, focuses on individual control of thoughts, actions, and motivations to achieve goals (Lin, 2018).

Interviews with lecturers (3.3) complement these findings by emphasizing self-regulation and cognitive strategies, the key dimensions that distinguish the latent profiles. Lecturers highlight the value of planning, the use of technologies (LMS, simulators), and hands-on activities for learning, which align with the strengths of Profile 0. Additionally, training in virtual environments facilitates voluntary control of brain activity, which is especially useful for students with difficulties in neuronal self-regulation. A notable example is *MindTrain*, a gamified system that integrates VR and mobile electroencephalography (EEG) to help students regulate their brain activity through relaxation and concentration techniques in immersive environments (Kosuru et al., 2019; Baqapuri et al., 2021).

The concept of ‘mental toughness’ and self-leadership emphasized by military instructors reflects an appreciation of resilience and self-efficacy—qualities prominent in Profile 0 but lacking in Profile 2. Interestingly, test anxiety and intrinsic motivation were the least mentioned dimensions in the interviews, despite being key factors in differentiating the profiles. However, the use of VR in learning presents challenges related to cognitive overload, particularly with visual and audiovisual material, with the former generating a higher cognitive load (Albus and Seufert, 2023). This suggests the need for greater focus on these aspects in teaching, particularly for Profile 2 students.

### 7.1 Implications and significance of the results

Differentiated learning profiles have direct implications for military pedagogy. First, the diverse profiles of military students need adapting instructional resources to maximize their potential. An example in immersive learning environments is the use of spherical video in virtual reality (SVVR). Through telepresence, SVVR has been shown to significantly impact self-regulation, self-efficacy, and attitudes toward learning—essential competencies in online education (Wu et al., 2021; Wang et al., 2023). Second, findings from the profile analysis and interviews emphasize the need to explicitly strengthen self-regulation and cognitive strategies in curricula. This could involve workshops, activities, and resources aimed at improving planning, information management, autonomous learning, and effective study strategies, particularly for students in Profiles 2 and 3, who exhibit low self-efficacy and motivation. Incorporating identified pedagogical and psychological traits into curricula can yield significant learning benefits. One model for optimizing immersive environments is the Cognitive-Affective Model of Immersive Learning (CAMIL), which examines six cognitive and psychological factors that influence learning outcomes in Immersive Virtual Reality (IVR) environments: interest, motivation, self-efficacy, embodied cognition—how the sensorimotor system perceives and interacts with the environment—cognitive load, and self-regulation (Makransky and Petersen, 2021) besides motivation, goal-setting, coping skills and behavioral activation (Oddo et al., 2021). Third, the emphasis on practical activities and technology use, as highlighted by teachers and embedded in the CPE structure of the programs, supports the continuation and refinement of these methodologies. However, it is crucial to ensure that these tools and approaches are accessible and beneficial to all student profiles, including those with lower self-efficacy or inconsistent learning patterns, which can be identified through diagnostic assessments. Fourth, motivational interventions should incorporate emotional regulation strategies (e.g., resilience training, mindfulness) into curricula and other activities that target motivational constructs.

Virtual Experiment Environments (VEEs), for example, have proven to be effective tools for laboratory class preparation, as they take into account students’ prior knowledge (Verstege et al., 2019). Fourth, the discrepancy between the minimal mention of anxiety and intrinsic motivation in interviews and their significance in the profiles highlights the need to raise teachers’ awareness of these emotional and motivational factors. In this context, feedback plays a crucial role in enhancing self-regulation (Butler and Winne, 1995) which in turn needs to adapt to immersive environments (Wang et al., 2024). Research on technological innovations highlight several differences between virtual reality (VR) feedback and traditional feedback in activities such as oral presentations. VR feedback addresses cognitive, behavioral, and attitudinal aspects, providing notable benefits in higher education (Van Ginkel et al., 2019).

Despite the advantages of virtual learning, physical isolation can negatively impact the educational experience by limiting social interaction and engagement. Research indicates that integrating virtual reality with self-regulated strategies, such as the Self-Regulated Strategy Development (SRSD) model, significantly enhances teacher-student interaction, learning self-regulation, and academic performance (Shen and Li, 2022). Strategies to foster intrinsic motivation and reduce test anxiety could also be integrated into teacher training and course design. This is particularly relevant in fields such as linguistics, business communication, sciences, humanities, English as a foreign language, and physical education, as it also enhances spatial attention and optimizes brain rhythms (Alhalangy and Abdalgane, 2023; Jeunet et al., 2020).

Additionally, collaboration in virtual environments can also be enhanced through interactions with virtual pedagogical agents (PAs). Systems, e.g., MetaTutor have demonstrated improvements in academic performance and student motivation, as learners tend to consistently follow the suggestions and feedback provided by these agents (Harley et al., 2018; Tinôco et al., 2021). This potential extends to other emerging technologies. An example is *MindTrain*, a gamified system that combines VR and mobile electroencephalography (EEG) to help students regulate their brain activity through relaxation and concentration techniques in immersive environments (Kosuru et al., 2019).

## 8 Conclusion

In conclusion, the mixed-method approach used in this study has given us a comprehensive and detailed view of how military students learn. By combining learning profiles, curriculum analysis, and teaching perspectives, this study reveals the complexity of military education and highlights the need for adaptive pedagogical approaches. The findings emphasize the importance of personalized teaching, enhanced self-regulation and cognitive strategies, optimized practical and technological methodologies, and greater attention to motivational and emotional factors. Although intrinsic motivation and test anxiety were among the least mentioned dimensions in the interviews, they emerged as key differentiators in the latent profiles, particularly for Profiles 2 and 3. This suggests a gap in teacher awareness regarding the emotional components of learning. To address this, we recommend the integration of emotional regulation strategies—such as resilience training, mindfulness, and structured emotional feedback—into curricula and training programs. Additionally, gamified platforms targeting motivation could be explored to complement traditional military pedagogies.

Technology can be seen as an integrated component that enhances the effectiveness of these recommendations. LMS platforms, flight and mechanical simulators, and other digital tools serve as valuable resources for adapting instruction to different student profiles. These technologies support self-regulation and cognitive strategies through interactive content and personalized feedback while enriching practical activities with immersive simulations. Proper selection and implementation of educational technologies, informed by a deep understanding of military student learning profiles, could be determinant in optimizing their training and preparing them for the challenges of a technologically advanced military environment.

Yet, this study has limitations that must be acknowledged. The specificity of the military context and the institutions analyzed restricts the direct applicability of these results to other educational settings. But hypothetically, as previously mentioned, military students do not fundamentally differ from their civilian counterparts. Although immersive technologies were discussed in interviews and document reviews, they were not directly measured via the MSLQ. Rather, they were explored qualitatively to understand contextual adoption.

The use of the MSLQ is based on self-assessment, which might potentially introduce biases. While valuable, the interviews reflect only the perspectives of a specific group of lecturers and exclude other perspectives. Future research could broaden the study’s geographic and institutional scope by incorporating a more diverse sample of higher education institutions. Conducting longitudinal studies to analyze the evolution of learning profiles throughout military training would too be highly valuable. Future studies should incorporate student voices through interviews or focus groups to gain a deeper understanding of their emotional and motivational challenges in adopting new technologies. Finally, assessing the impact of personalized pedagogical interventions tailored to each profile’s specific needs would be essential for applying these findings in practice. The use of qualitative methods among military students, such as interviews or focus groups, would provide deeper insights into this phenomenon from their perspective.

## Data Availability

The original contributions presented in the study are included in the article/supplementary material, further inquiries can be directed to the corresponding authors.

## Glossary

MSLQ: Motivated Strategies for Learning Questionnaire
IVR: Immersive Virtual Reality
LMS: Learning Management System
VR: Virtual Reality
AR: Augmented Reality
CAQDAS: Computer-Assisted Qualitative Data Analysis Software
CD: Contact with the Teacher (Contacto Docente)
CPE: Experiential Practical Component (Componente Práctico Experimental)
CA: Autonomous Component
SRL: Self-Regulated Learning
PBL: Problem-Based Learning
ESPE: Universidad de las Fuerzas Armadas ESPE
VA: Group VA
OE: Group OE
VM: Group VM
OM: Group OM
OA: Group OA
AI: Artificial Intelligence
COVID-19: Coronavirus Disease 2019
TC: Teacher Contact
EL: Experiential Learning
AL: Autonomous Learning
SPSS: Statistical Package for the Social Sciences
