# Correction: Establishing the foundation for technology adoption: profiles of military students in the digital age

**DOI:** 10.3389/fpsyg.2025.1680341

**Published:** 2025-09-08

**Authors:** Andrea Luna, Mauro Ocaña, Javier Rodríguez-Moreno, Ana María Ortíz-Colón

**Affiliations:** ^1^Universidad de las Fuerzas Armadas ESPE, Sangolquí, Ecuador; ^2^University of Jaén, Jaén, Spain

**Keywords:** latent profile analysis, MSLQ, learning profiles, educational technology, military students, self-regulated learning

Affiliation “Universidad de las Fuerzas Armadas ESPE, Sangolquí, Ecuador” was erroneously given as “Human and Social Sciences, University of the Armed Forces (ESPE), Sangolquí, Ecuador, Quito-Conocoto, Ecuador” for authors Andrea Luna and Mauro Ocaña.

In Affiliation 2, the department was erroneously included. The original version appears below:

Department of Human and Social Sciences, University of Jaén, Jaén, Spain

The corrected version appears below:

University of Jaén, Jaén, Spain

The original version of this article has been updated.

